# Deep learning-based identification of esophageal cancer subtypes through analysis of high-resolution histopathology images

**DOI:** 10.3389/fmolb.2024.1346242

**Published:** 2024-03-19

**Authors:** Syed Wajid Aalam, Abdul Basit Ahanger, Tariq A. Masoodi, Ajaz A. Bhat, Ammira S. Al-Shabeeb Akil, Meraj Alam Khan, Assif Assad, Muzafar A. Macha, Muzafar Rasool Bhat

**Affiliations:** ^1^ Department of Computer Science, Islamic University of Science and Technology, Awantipora, India; ^2^ Human Immunology Department, Research Branch, Sidra Medicine, Doha, Qatar; ^3^ Department of Human Genetics-Precision Medicine in Diabetes, Obesity and Cancer Program, Sidra Medicine, Doha, Qatar; ^4^ DigiBiomics Inc, Mississauga, ON, Canada; ^5^ Department of Computer Science and Engineering, Islamic University of Science and Technology, Awantipora, India; ^6^ Watson-Crick Centre for Molecular Medicine, Islamic University of Science and Technology, Awantipora, India

**Keywords:** histopathology, deep learning, machine learning, transfer learning, image processing, whole slide image, patching, normalization

## Abstract

Esophageal cancer (EC) remains a significant health challenge globally, with increasing incidence and high mortality rates. Despite advances in treatment, there remains a need for improved diagnostic methods and understanding of disease progression. This study addresses the significant challenges in the automatic classification of EC, particularly in distinguishing its primary subtypes: adenocarcinoma and squamous cell carcinoma, using histopathology images. Traditional histopathological diagnosis, while being the gold standard, is subject to subjectivity and human error and imposes a substantial burden on pathologists. This study proposes a binary class classification system for detecting EC subtypes in response to these challenges. The system leverages deep learning techniques and tissue-level labels for enhanced accuracy. We utilized 59 high-resolution histopathological images from The Cancer Genome Atlas (TCGA) Esophageal Carcinoma dataset (TCGA-ESCA). These images were preprocessed, segmented into patches, and analyzed using a pre-trained ResNet101 model for feature extraction. For classification, we employed five machine learning classifiers: Support Vector Classifier (SVC), Logistic Regression (LR), Decision Tree (DT), AdaBoost (AD), Random Forest (RF), and a Feed-Forward Neural Network (FFNN). The classifiers were evaluated based on their prediction accuracy on the test dataset, yielding results of 0.88 (SVC and LR), 0.64 (DT and AD), 0.82 (RF), and 0.94 (FFNN). Notably, the FFNN classifier achieved the highest Area Under the Curve (AUC) score of 0.92, indicating its superior performance, followed closely by SVC and LR, with a score of 0.87. This suggested approach holds promising potential as a decision-support tool for pathologists, particularly in regions with limited resources and expertise. The timely and precise detection of EC subtypes through this system can substantially enhance the likelihood of successful treatment, ultimately leading to reduced mortality rates in patients with this aggressive cancer.

## Introduction

Esophageal cancer (EC), which has seen a concerning rise in prevalence over the past 4 decades (Napier et al., 2014), now ranks as the eighth most common cancer and the sixth leading cause of cancer-related mortality worldwide (Pakzad et al., 2016). This aggressive disease presents a formidable challenge, with 5-year survival rates between 15% and 25% (Pennathur et al., 2013). In 2020 alone, approximately 600,000 new EC cases were reported, tragically resulting in an estimated 540,000 fatalities (Sung et al., 2021). The origins of this malignancy typically lie in the mucosa, the innermost layer of the esophagus, and from there, it can infiltrate various vital organs, including the stomach, liver, lungs, chest, blood vessels, and lymph nodes. EC comprises two primary subtypes: Esophageal Squamous Cell Carcinoma (SCC) and Adenocarcinoma (ADC), along with a handful of relatively uncommon small-cell carcinomas like melanoma, sarcoma and lymphoma (Abbas and Krasna, 2017; Yang et al., 2020).

In medical diagnostics, histopathological image analysis is the gold standard for cancer diagnosis (Gurcan et al., 2009; Tomaszewski, 2021). Histopathological images provide a comprehensive view of diseases, capturing many cytological features that furnish detailed diagnostic insights. These features encompass heightened mitotic activity, cellular enlargement, nuclear hyperchromasia, irregular nuclear chromatin distribution, prominent and sizable nucleoli, cellular and nuclear pleomorphism, increased cellularity, abnormalities in nuclear membranes, cellular discohesiveness, and the presence of tumor necrosis in the background, also known as tumor diathesis. Unlike mammography or CT scans, these cytological characteristics are not easily discernible through other imaging techniques (Al-Abbadi, 2011). Nevertheless, conventional methods employed to analyze histopathology whole-slide images (WSI) have limitations, notably in time consumption and observer variability. Traditionally, pathologists scrutinize tissue samples under a microscope, having stained them with Haematoxylin and Eosin (H&E) to enhance tissue organ contrast (He et al., 2012). However, this labour-intensive visual inspection and subjective interpretive process require pathologists to examine multiple regions of interest within each slide.

Furthermore, the inherent subjectivity in visual interpretation can introduce variations among different observers, leading to inconsistent diagnoses and assessments (Allison et al., 2014). Hence, a compelling need exists for more efficient and objective approaches to WSI analysis, such as advanced digital pathology techniques that leverage image analysis algorithms, artificial intelligence, and machine learning. These cutting-edge methods aim to automate and streamline the analysis process, diminishing the reliance on manual examination and enhancing efficiency, consistency, and accuracy in evaluating histopathology WSI.

Artificial intelligence (AI) and deep learning (DL) diagnostic systems are becoming increasingly popular, opening up new possibilities in image analysis (Guo et al., 2023; Muller et al., 2023; Wang et al., 2023; Xiao et al., 2023). By harnessing shape and texture attributes alongside higher-order spatial features that capture intricate pixel-level relationships, these systems elevate images into high-dimensional features, vastly enhancing their capability for detection and classification (Kumar et al., 2012). While feature extraction-based classification systems, which rely on handcrafted features to characterize cancer subtypes, have seen advancements, researchers increasingly recognize the potency of deep neural networks (DNN) for automatic feature extraction and comprehensive visual analysis (Kermany et al., 2018; Shimizu and Nakayama, 2020; Zhou et al., 2020). Unlike traditional manual approaches, deep learning’s automatic feature extraction through multiple non-linear transformation layers empowers it to capture a broad spectrum of generalizable and high-level features, particularly suited for intricate tasks like medical image analysis. Remarkably, both DL and machine learning (ML) have been deployed in intriguing ways for the diagnosis, prognosis prediction (Tran et al., 2021), and classification of various cancer types, utilizing diverse imaging modalities such as histopathology, CT scans, and MRI imaging. Their ability to handle vast medical data and discern complex patterns promises to revolutionize cancer diagnosis and treatment. In the context of EC patients, correct staging, treatment planning, and prognostication are pivotal for improving patient outcomes and survival rates (Huang et al., 2022). Unfortunately, EC is frequently diagnosed at advanced stages, owing to its non-specific early symptoms and the challenge of accessing the esophagus, resulting in a bleak prognosis. Thus, the importance of early detection cannot be overstated, as it holds the key to timely interventions, exploration of a broader range of treatment options, and, ultimately, better long-term survival for patients.

A recent investigation (Wagner and Cosgrove, 2023) utilized ResNet-18 for endometrial subtype classification, employing Tissue level annotation of WSIs from the (TCGA-UCEC) dataset. This study achieved the best AUC of 0.82 in predicting the subtypes of cancer. In another research (Zhao et al., 2023), a DL model based on ResNet50, utilizing 523 annotated whole-slide images, demonstrated the capability to classify invasive non-mucinous lung adenocarcinoma subtypes with an AUC of 0.80. A study has employed transfer learning to extract features, subsequently utilizing various machine learning models for classification, achieving an accuracy of 0.93 (Kumar A and Mubarak, 2022). Another study presents a two-stage deep transfer learning method for subclass classification in histopathology images, overcoming limitations in traditional patch-based approaches. While employing patch-based classification, the results are aggregated for slide-level classification. The study attains automatic classification of epithelial ovarian carcinoma WSIs with an accuracy of 0.87 (Wang et al., 2020).

Contemporary studies on esophageal cancer (EC) subtype classification predominantly rely on patch-level annotations, introducing different challenges. The reliance on such annotations has been associated with inter-observer variability and limitations in capturing the comprehensive spatial and morphological distinctions inherent in esophageal cancer tissues (Paul and Mukherjee, 2015). The imperative for direct pathologist involvement in the annotation process not only imposes heightened time and resource demands but also intensifies the potential for divergent annotation practices. The repeated emphasis on direct pathologist involvement extends the temporal and resource investment and underscores the susceptibility to variations in annotation practices. These variations further challenge the integration and comparability of results across diverse research studies, hindering the establishment of a cohesive and standardized understanding of EC subtypes (Evans et al., 2023).

Addressing this gap, we aimed to develop an advanced DL-based automated diagnostic system, leveraging state-of-the-art deep learning techniques for feature engineering and machine learning, to accurately classify different subtypes of esophageal cancer using H&E images of the esophagus. While adhering to fundamental predictive modelling procedures, we have introduced an innovative approach by incorporating a median-based technique to effectively address the inherent variability in the number of tumor tiles across Whole Slide Images (WSIs) and demonstrate resilience to outliers, enhancing the stability and reliability of our model. We trained our model on a dataset comprising 42 high-resolution WSI and subsequently validated it using 17 additional images.

## Methodology

### Data collection

**FIGURE 1:**
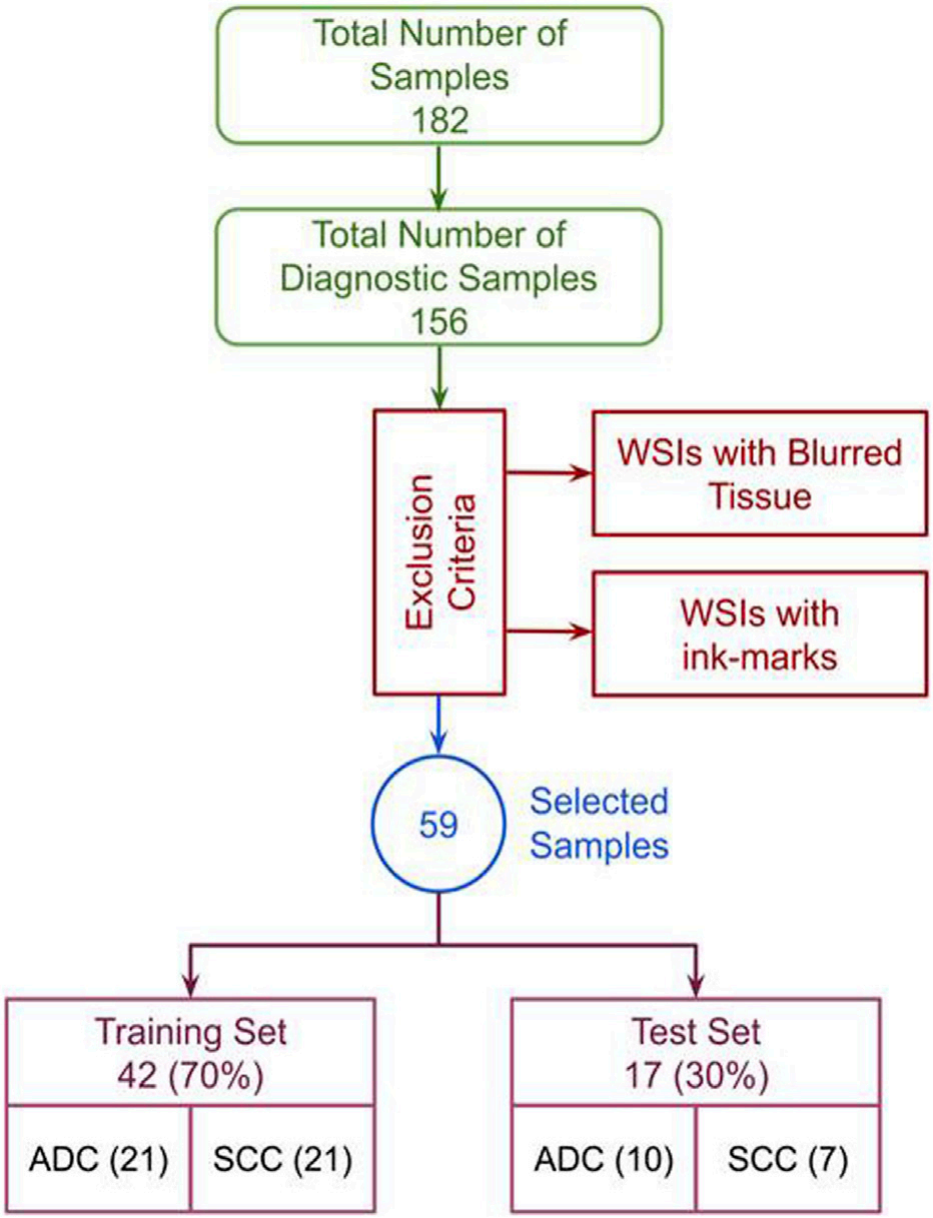
Exclusion criteria: The complete data selection process, incorporating exclusion criteria and showcasing the data distribution in train and test sets.

The Cancer Genome Atlas Esophageal Carcinoma Collection (TCGA-ESCA) cohort comprises diagnostic and tissue slides for 182 subjects. Diagnostic slides, which provide detailed information about tissue phenotypic heterogeneity (Chen et al., 2022), were exclusively focused upon due to their significance in histologic analysis. This study did not consider tissue slides, often used for genomic analysis (Gutman et al., 2013). As such, only 156 subjects with diagnostics slides available in TCGA-ESCA were selected, as shown in Figure 1. Following rigorous filtering criteria by excluding the WSIs with poor visual quality, extensive blurring, abnormal staining, and ink marks. Finally, 59 high-quality diagnostic WSIs were obtained from the TCGA-ESCA cohort. This curated dataset, consisting of H&E stained WSIs categorized into SCC and ADC, ensured consistency and quality for subsequent research and analysis. Significantly, the TCGA cohort’s open accessibility without authentication requirements greatly facilitated our investigative activities.

### Preprocessing of histopathology images

Training DNN and ML models directly on entire gigapixel H&E WSI presents substantial computational challenges with current standard computing resources. We adopted strategies such as tiling and down-sampling (Srinidhi et al., 2021). High-resolution images are broken down into smaller, more manageable patches. Within these WSIs, there were both informative and non-informative regions. The Canny edge detection algorithm was employed for its ability to differentiate and outline tissue boundaries, leveraging its capability to identify noisy edges effectively. The method offers criteria of strong edge detection, precise localization, and single response, making it a suitable choice for our purpose (Canny, 1986). Subsequently, masks were generated to demarcate the boundary between the background and the tissue (foreground) using a graph-based segmentation method. The method was chosen due to its adeptness in handling pixel intensity variations, ensuring resilience against challenges posed by staining and tissue characteristic variations, and fostering consistent and reliable boundary demarcation (Felzenszwalb and Huttenlocher, 2004). These mask images were then organized into a grid of tiles based on specified dimensions to form patches. Patches comprising background information were discarded, while those primarily containing tissue (foreground) information were retained for further analysis. Various techniques, including random sampling, Otsu, and adaptive thresholding, can be used to eliminate background tiles (Otsu, 1979; Kalton, 2011; Roy et al., 2014). We utilized the Otsu method, a widely used thresholding algorithm (Goh et al., 2018; Huang et al., 2021; Singh et al., 2021), to discard unwanted background images from the generated patch set due to its thresholding capability for varying image characteristics, allowing for optimal discrimination between tissue and background in an unsupervised manner. Tiles containing less than 10% tissue were excluded and classified as background by setting the threshold to 0.1. Histology images were preprocessed using PyHIST (Muñoz-Aguirre et al., 2020), a TIFF/SVS file format segmentation tool. Nonoverlapping patches measuring 512 × 512 pixels were extracted from each WSI, and H&E images with a magnification greater than ×20 were only considered for this study.

### Data normalization

Histopathology images exhibit inherent color variations stemming from differences in staining concentration, varied equipment usage, and occasional inconsistencies in tissue sectioning. These color disparities within WSI introduce increased variability into the training data, potentially impacting the model’s efficacy during training. Various methods exist for image color normalization, including the Reinhard Algorithm (RA) (Reinhard et al., 2001), Histogram Specification (Hussain et al., 2018), and Structure-Preserving Color Normalization (Vahadane et al., 2016), with RA notably demonstrating superior performance in DL models. The RA method computes a chosen reference image’s mean and standard deviation. During the color correction process, the color characteristics of one image are aligned with those of another. Careful consideration was given to the reference image selection, as normalization outcomes can be influenced if the chosen reference image exhibits darker colors (Roy et al., 2021). The application of Reinhard color normalization to our dataset is based on established practices in the literature (Lakshmanan et al., 2019; Qu et al., 2021), demonstrating its superior performance compared to other techniques. This normalization method effectively mitigated the complexity of the extracted features, thereby enhancing the overall quality of our analysis.

### Model training and evaluation

**FIGURE 2:**
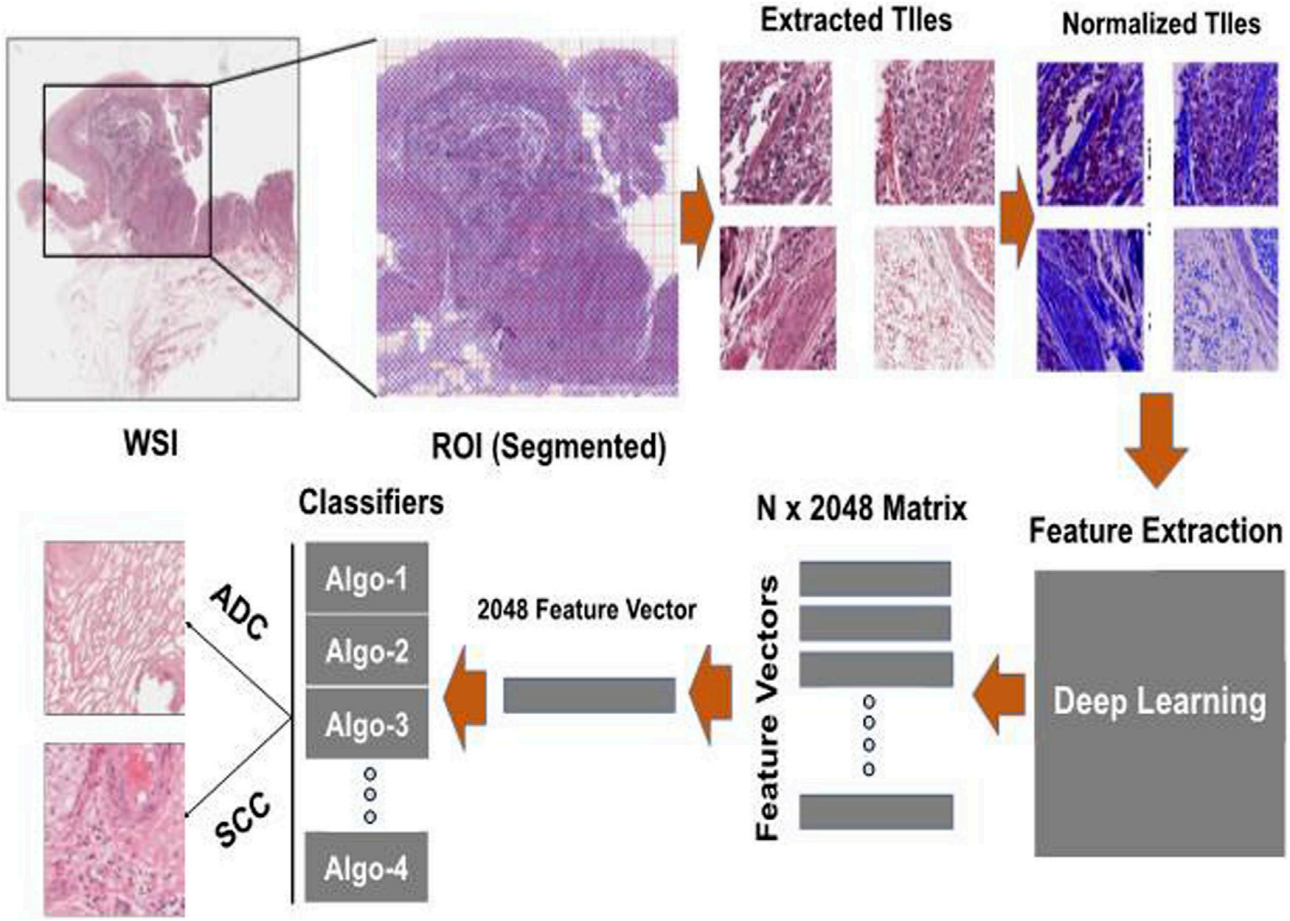
Experimentation pipeline: The process involves taking each color-normalized tile and passing it through a pre-trained model to obtain a 2048-dimensional feature vector. These feature vectors from all tiles belonging to the same patient are then combined into a single feature vector of size 2048 and used as input for various machine-learning models to make predictions about esophageal cancer (EC) subtypes.

We employed a two-step approach for classification, as outlined in Figure 2. In the initial step, we performed feature extraction on all the tiles within the WSI using a pre-trained CNN (ResNet101) (He et al., 2016). This process generated a 2048-dimensional feature vector for each tile in the WSI. Consequently, we constructed a feature matrix of size N x 2048 for each subject, where N represents the number of tumor tiles within the WSI. This variable significantly differs across WSIs. The variance in the number of patches generated during the tiling process impacts the model’s prediction efficiency. To address this, we calculated the median of the stacked feature vectors from all the WSI tiles along the vertical axis, thereby creating a single feature vector with a size of 2048. These feature vectors were subsequently input into various classification algorithms to predict the EC subtypes. The experimentation was conducted on an NVIDIA Tesla V100 GPU with 32 GB memory, boasting 15,360 CUDA Cores and a processing power of 2.976 TFLOPS. On average, normalization per tile required 0.25 s, and Feature extraction exhibited an average time of 0.03 s per tile. These metrics underscore the computational efficiency of the algorithms on the specified high-performance GPU architecture. We employed performance metrics such as Accuracy, AUC, Recall, F1-score, and Precision to assess the proposed model’s efficacy in classifying EC subtypes. Accuracy provides a comprehensive assessment of correctness in both classes, particularly effective in balanced class distributions. AUC evaluates the model’s discriminatory ability across various thresholds. Recall quantifies the model’s efficacy in identifying positive instances out of total positive instances, which is crucial in scenarios prioritizing sensitivity. Precision measures the accuracy of actual positive predictions, offering insights into the model’s specificity. Lastly, the F1 score strikes a balance between precision and recall, furnishing the evaluation of overall model performance in classification tasks.

During the model training and evaluation, we utilized a Feed-Forward Neural Network (FFNN) configured with only two layers. The first layer, with a size of 2048, was designed to accept the input feature vector, preserving compatibility with the size of the extracted features obtained during the feature extraction process using pretrained models. The second layer was employed for classification, strategically reintegrating the last layer of the pretrained model, which was initially removed during the feature extraction process. This approach allows for an efficient utilization of the deep hierarchical representations learned by the model. The choice of two dense layers with Rectified Linear Unit (ReLU) activation function (Agarap, 2018), a learning rate of 10^–3^ over 800 epochs, and the Adam as optimizer (Kingma and Ba, 2015) was opted due to the ability of DL to complex information through multiple layers (Bebis and Georgiopoulos, 1994). In addition to FFNN, we utilized various machine learning classifiers for comparative analysis following the previous studies (Singh et al., 2023; Saxe and Berlin, 2015). AdaBoost, an ensemble learning method, was chosen for its ability to combine weak classifiers, such as decision trees, to form a robust classifier (Freund and Schapire, 1997). Decision Tree, a non-parametric algorithm, was also employed for its tree-like structure and adaptability to handle noise and missing data (Cycles, 1989). Random Forest, another ensemble method was selected to enhance classification performance by combining multiple decision trees through bootstrap sampling and feature subsetting (BREIMAN, 2021). Logistic Regression, a binary classification algorithm, served as a widely used and interpretable model, estimating probabilities for esophageal carcinoma subtypes based on input feature vectors (Peng et al., 2002). Finally, the Support Vector Classifier (SVC), known for its robustness and versatility in handling both linear and non-linearly separable data, was applied for effective classification between esophageal cancer subtypes (Busuttil, 2003). Each model brought a unique strategy to the analysis, contributing to a comparative analysis of our proposed approach, which provides valuable insights into the suitability of various machine learning algorithms.

## Results

### High-resolution histopathological dataset of ADC and SCC

**FIGURE 3:**
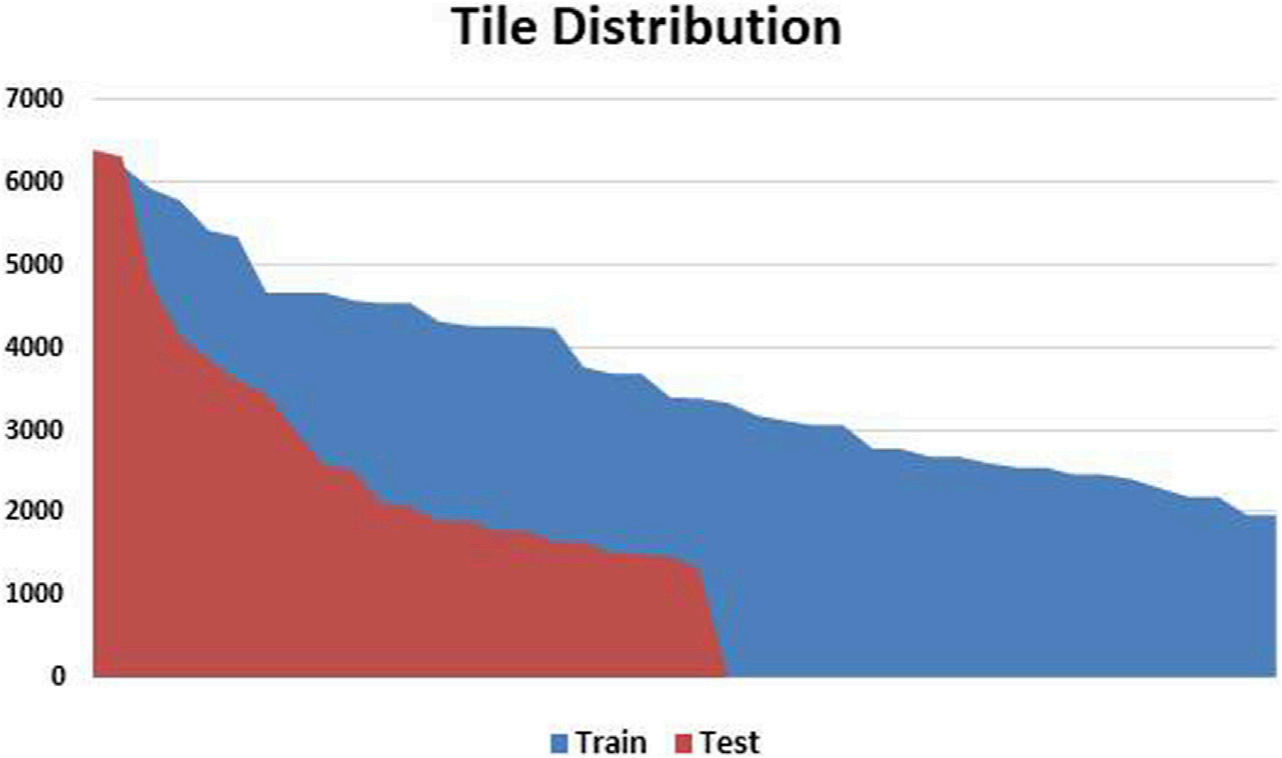
Patch Variation and Distribution: This figure visually presents the patch variation after preprocessing, showing the data distribution between training and testing sets. Patch counts range from as high as 6,000 patches per slide to as low as 1,200 patches per slide. The variation is handled by calculating the median of all the features per WSI to form a single feature vector.

Following a meticulous qualitative analysis that excluded small-sized images and those with significant blurring artefacts and ink marks, we systematically organized a dataset comprising 59 high-resolution histopathological H&E stained images of ADC and ESCC. This dataset was then divided into distinct training and testing sets, maintaining a balanced 70:30 ratio. After extracting WSI tiles and carefully selecting tumor tiles, the training and testing sets for EC comprised 191,798 and 48,665 tiles, respectively. The distribution of these tiles is visually depicted in Figure 3 for both the train and test sets. Moreover, the demographic and other characteristics of all patients included in our experimentation are summarized in Table 1.

**TABLE 1:** Patient characteristics on the 59 cases from TCGA - ESCA cohort.

Characteristics		Data set	
		ADC	SCC
Gender	Male	26	25
	Female	5	3
Age	≤60	9	21
	>60	22	7
Vital Status	Dead	14	8
	Alive	17	20
Alcohol History	Yes	26	19
	No	5	9
Location	Lower Third	29	11
	Middle Third	-	12
	Upper third	2	3
	Unspecified Location	-	2

### Enhancing digital pathology by data-preprocessing

**FIGURE 4:**
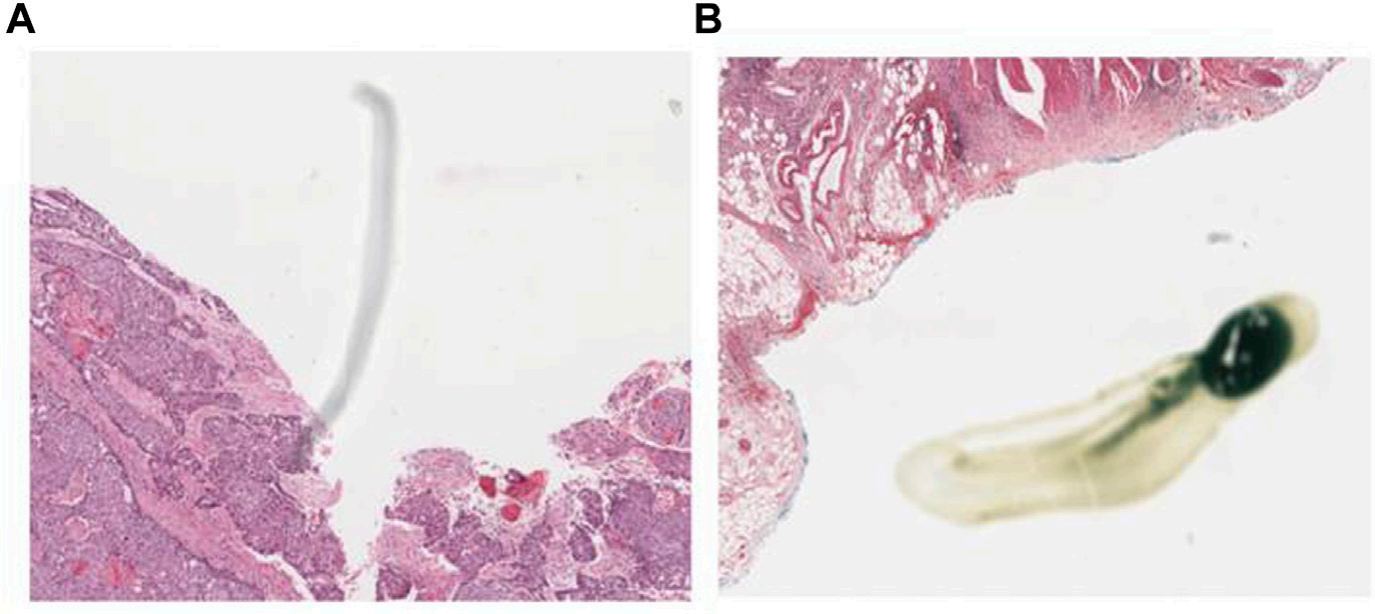
WSI Noise and Artefacts: **(A)** an example of noise in WSI (random or unwanted variations in image data), **(B)** an example of artefact in the Whole Slide Image of our dataset. (Artefacts can include staining artefacts, tissue folding or tearing, scanner artefacts, or any other unintended alterations that may occur during slide preparation, scanning, or digitization).

**FIGURE 5:**
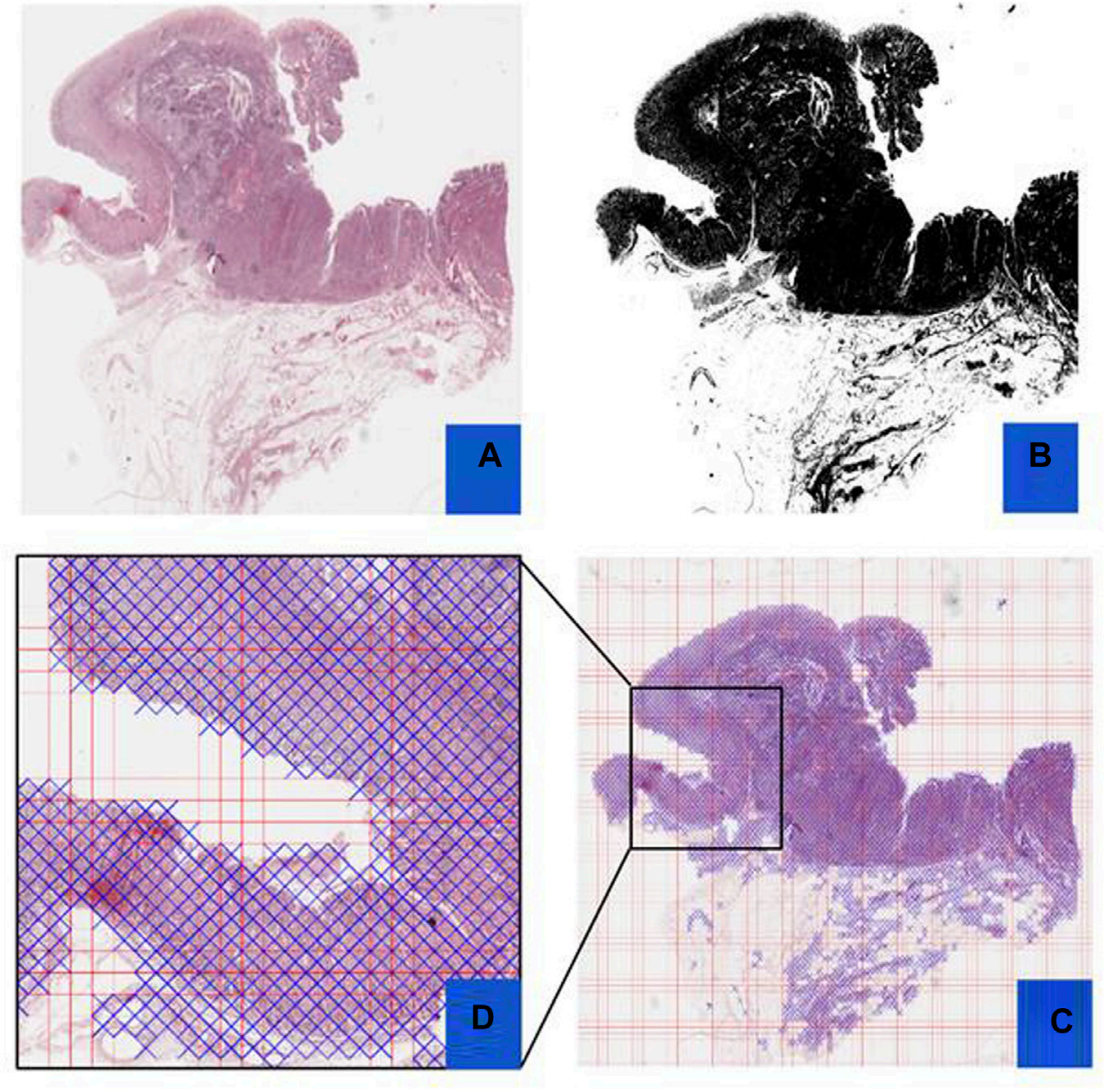
Preprocessing Steps: This figure outlines the preprocessing steps using PyHIST. Panel **(A)** displays an H&E WSI from TCGA, **(B)** illustrates a mask generated during preprocessing, **(C)** presents a Mask Grid created for patching WSI, and **(D)** shows a zoomed view of tiles selected from the grid.

**FIGURE 6:**
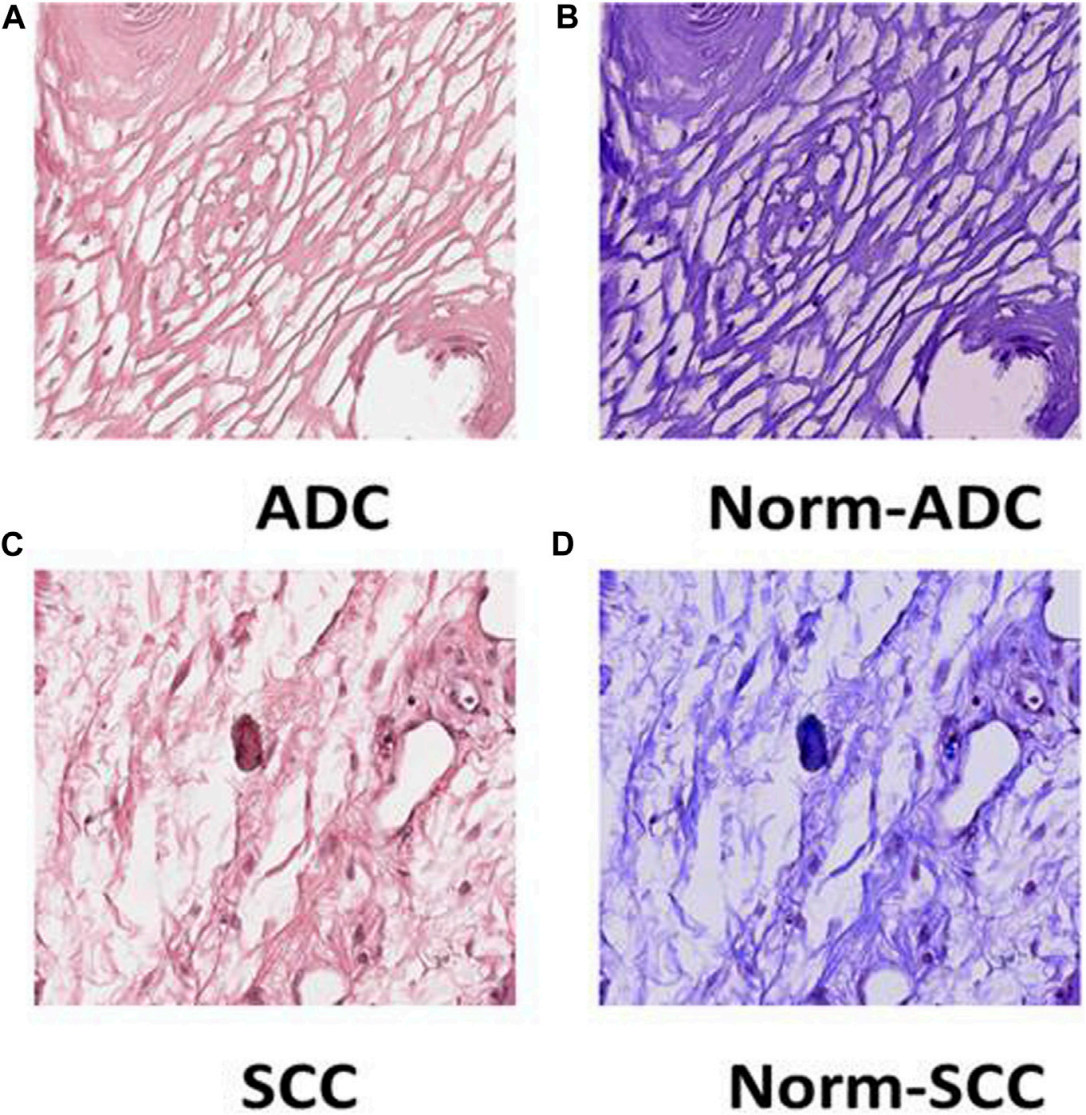
Tile Transformation: The figure exhibits the normalization process for ADC and SCC tiles. Panel **(A)** displays an original tile of ADC WSI after patching, **(B)** shows the ADC tile after normalization, **(C)** presents an original tile of SCC WSI after patching, and **(D)** illustrates the SCC tile after normalization.

**FIGURE 7:**
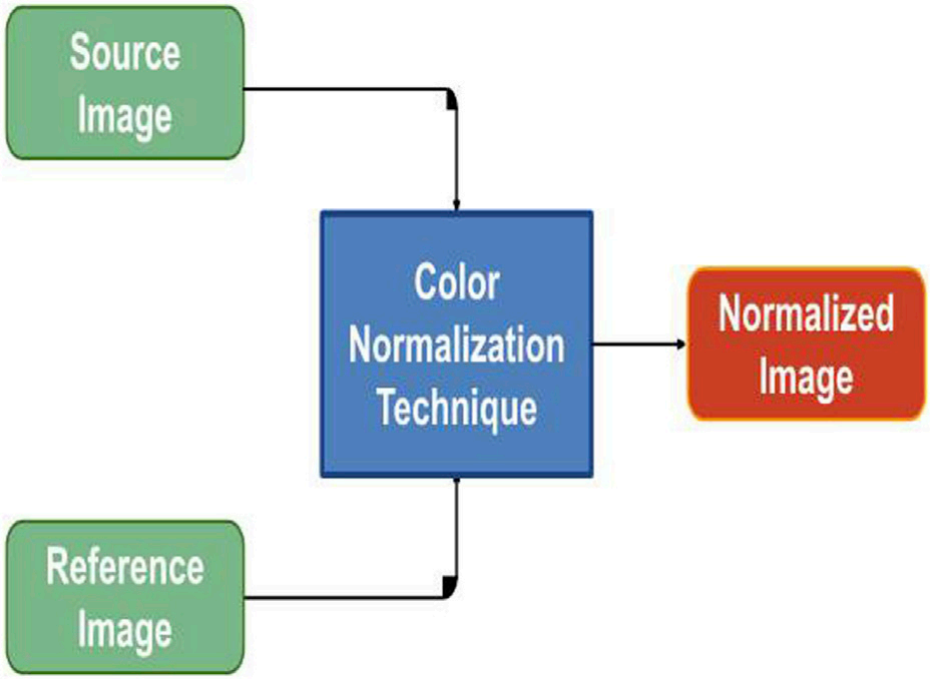
Color Normalization Flowchart: The figure provides a flow chart representing the color normalization process for images with different color variations. This process enhances the consistency of color across images, ensuring uniformity in the dataset.

Preprocessing of digital pathology played a crucial role in addressing differences among images, including variations in color, illumination, and imperfections like artefacts or noise (Figure 4). These variations and imperfections can be effectively minimized or corrected by applying preprocessing techniques, ensuring more consistent and reliable image quality across diverse datasets. The advantages of digital pathology still need to be increased to overcome its limitations: WSI requires considerably large storage volumes (since each image can need 2–3 GB). Moreover, as depicted in Figure 5, these WSIs exhibit undesirable blank backgrounds, which were eliminated during this stage. Despite efforts toward standardization, attaining impeccable color calibration across all samples remains challenging (Lyon et al., 1994). This challenge drives the adoption and implementation of color normalization techniques, which have led to increased efficiency in our model. Figure 6 provides a visual difference before and after color normalization, and the general normalization process is shown in (Figure 7).

### Performance evaluation and comparative analysis

**FIGURE 8:**
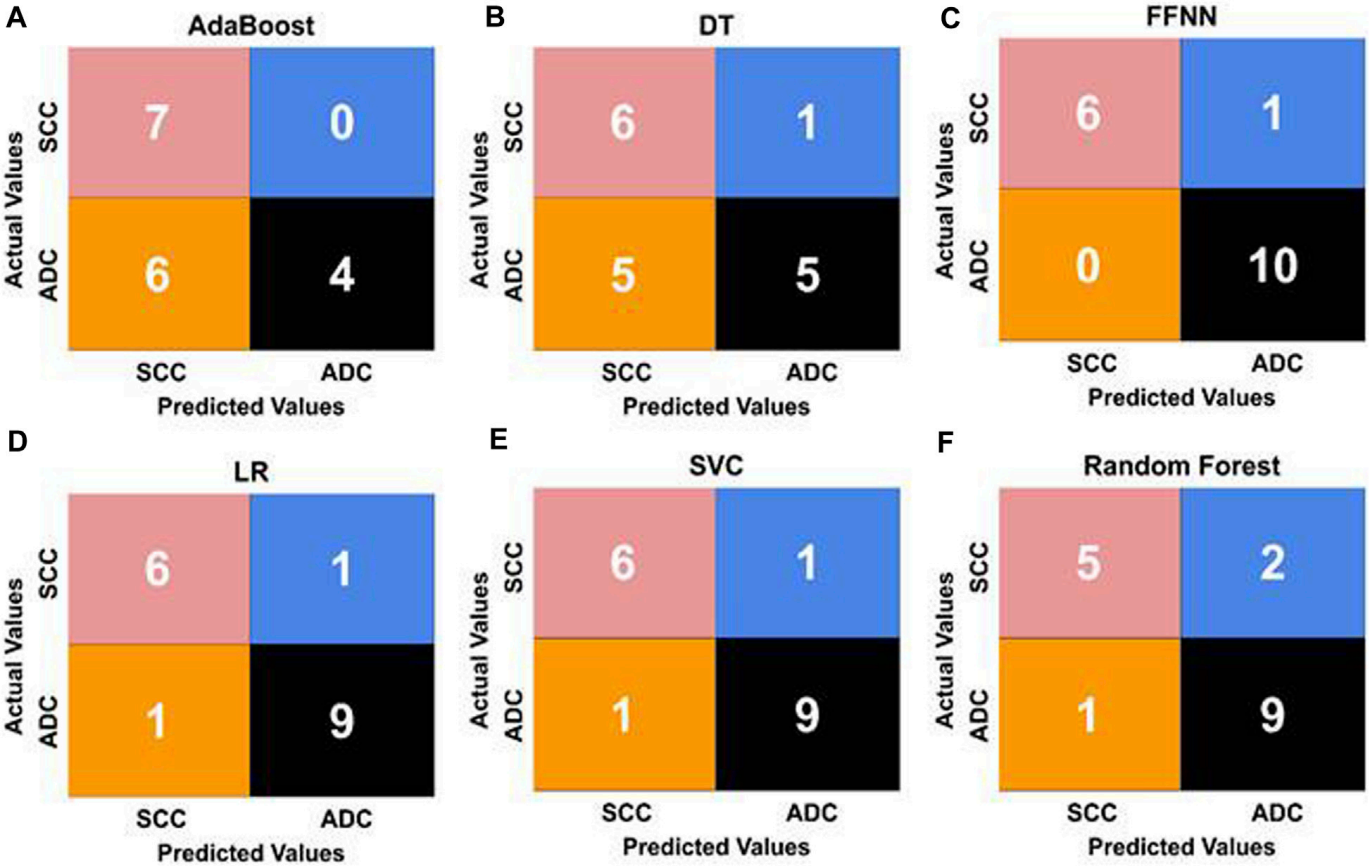
Confusion matrices illustrating the performance of five ML classifiers [represented as **(A, B, D, E, F)**]. **(C)** shows the confusion matrix of the FFNN classifier with the best values for classifying esophageal cancer subtypes. The matrices display the true positive (TP), true negative (TN), false positive (FP), and false negative (FN) values for each model, offering insights into their predictive capabilities and error distributions.

We utilized confusion matrices, as illustrated in Figure 8, to assess the overall accuracy of our classification models. Calculating recall, precision, and F1 score from the confusion matrix values provided a comprehensive evaluation of model performance for both SCC and ADC classes. This approach aligns with established practices, reflecting evaluation matrices utilized in previous studies (Le and Ou, 2016; Le et al., 2017). The FFNN stands out as the top-performing model, achieving an impressive classification rate of 86% for SCC patients and 100% for ADC patients in the validation set. Our feature-based classification method successfully demonstrated the use of machine learning in accurate classification of esophageal squamous cell carcinoma and adenocarcinoma. The SVC and LR models also demonstrated strong performance, achieving prediction accuracies of 86% for SCC patients and 90% for ADC patients. The RFC provided acceptable predictions of 71% for SCC patients and 90% for ADC patients. AdaBoost achieved a perfect prediction rate (100%) for SCC patients but presented the lowest performance of 40% only for ADC patients. The Decision Tree model also exhibited the least effectiveness, with correct classification rates of 85% for SCC patients and 50% for ADC patients.

Due to the large size, WSIs were divided into smaller patches for analysis, and image features were extracted at a ×20 magnification level. The results from different ML classification methods, utilizing identical methodology and data distribution, were compared while maintaining the training and test ratio. These classifiers use the feature vectors of size N x 2048 as input, extracted using ResNet101. The evaluation considered essential performance metrics such as precision, recall, F1-score, accuracy, and AUC. The results in Table 2 demonstrated that the feed-forward neural network (FFNN) model outperformed other ML models with an accuracy of 94.11% and an AUC score of 92%.

**TABLE 2:** Results of WSI classification.

Feature extraction	Algorithms	Precision		Recall		F1-score		ACC	AUC
		SCC	ADC	SCC	ADC	SCC	ADC		
Resnet101	SVC	0.86	0.9	0.86	0.9	0.86	0.9	0.88	0.87
	LR	0.86	0.9	0.86	0.9	0.86	0.9	0.88	0.87
	DT	0.55	0.83	0.86	0.5	0.67	0.62	0.64	0.68
	AdaBoost	0.54	1	1	0.4	0.7	0.57	0.64	0.7
	RFC	0.6	0.86	0.86	0.6	0.71	0.71	0.82	0.72
	FFNN	1	0.91	0.86	1	0.92	0.95	**0.94**	**0.92**
InceptionV3	SVC	0.75	0.89	0.86	0.80	0.80	0.84	0.82	0.82
	LR	0.70	1.0	1.0	0.70	0.82	0.82	0.82	0.85
	DT	0.67	0.73	0.57	0.80	0.62	0.76	0.71	0.68
	AdaBoost	0.71	0.80	0.71	0.80	0.71	0.80	0.76	0.75
	RFC	0.67	0.73	0.57	0.80	0.62	0.76	0.71	0.68
	FFNN	0.67	0.88	0.86	0.70	0.75	0.78	0.76	0.77
Resnet50	SVC	0.80	0.75	0.57	0.90	0.67	0.82	0.76	0.73
	LR	0.62	0.78	0.71	0.70	0.67	0.74	0.71	0.70
	DT	0.57	0.70	0.57	0.70	0.57	0.70	0.65	0.63
	AdaBoost	0.67	0.88	0.86	0.70	0.75	0.78	0.76	0.77
	RFC	0.67	0.73	0.57	0.80	0.62	0.76	0.71	0.68
	FFNN	0.75	0.89	0.86	0.80	0.80	0.84	0.82	0.82

Similarly, the SVC and LR models displayed comparable performance, achieving an accuracy of 88% and an AUC of 0.87, surpassing the performance of the remaining models. Notably, the DT and AdaBoost models exhibited the lowest accuracy at 64%. We observed that while FFNN outperforms all other algorithms in terms of accuracy, the imbalance in the dataset was better handled by SVC and LR, where the difference in recall between SCC and ADC is the least, showing that both classes have a similar number of misclassifications and accurate classifications. These metrics offer a holistic assessment of the model’s performance, highlighting its strengths and weaknesses. Such insights are invaluable for refining and advancing classification models in subsequent studies. Additionally, hyperparameters were carefully tuned using a grid search approach in our model training process, except for FFNN. They were optimized for each model to achieve the best performance on the test set (Table 3).

**TABLE 3:** Hyperparameters settings for selected models.

Model	Hyperparameter	Search space	Best value
AdaBoost	max_depth	[2, 11]	6
	min_samples_leaf	[5, 10]	10
	learning_rate	[0.01, 0.1]	0.1
	n_estimators	[10, 50, 250, 1,000]	50
Decision Tree (DT)	max_features	[‘auto’, ‘sqrt’, ‘log2']	auto
	ccp_alpha	[0.1, 0.01, 0.001]	0.01
	criterion	[‘gini’, ‘entropy']	gini
Random Forest (RF)	max_depth	[10, 15]	11
	max_features	[0, 14]	4
Logistic Regression (LR)	C	[0.1, 1, 10, 100]	0.1
	penalty	[‘l1', ‘l2']	l1
	solver	[‘newton-cg’, ‘lbfgs’, ‘liblinear']	liblinear
Support Vector Classifier (SVC)	C	[0.1, 1, 10, 100]	0.1
	kernel	[‘rbf’, ‘linear’]	linear
	gamma	[‘scale’, ‘auto']	scale

For a comparative analysis (refer to Table 2) involving transfer learning, we employed pre-trained InceptionV3 and ResNet50 models for feature extraction. The results showed that our proposed architecture outperformed other models, utilizing ResNet101 for feature extraction. The accuracy of our proposed model was 0.94, surpassing the best classification accuracy of 0.82 achieved by the other two models, thereby establishing the suitability of ResNet101 for the classification of esophageal cancer into Adenocarcinoma and Squamous Cell Carcinoma from histopathology images.

## Discussion and conclusion

Manual histopathological diagnosis of esophageal cancer subtypes poses challenges, emphasizing the need for a sophisticated and automated classification system. Our study adopts a tissue-level diagnostic approach leveraging deep learning and machine learning techniques to construct a reliable model, aiming to alleviate the limitations associated with manual diagnosis, providing a more efficient and objective solution.

We precisely curated the dataset TCGA-ESCA, ensuring consistency and quality while addressing potential variations in size, color intensities, and equipment usage. The preprocessing phase involved strategic techniques such as tiling, down-sampling, and edge detection to manage computational challenges and enhance the model’s efficiency. These methods facilitated the extraction of informative patches from high-resolution whole-slide images (WSI) for further analysis. Moreover, a data normalization technique was adopted, where inherent color variations in histopathology images are addressed using the Reinhard Algorithm. This normalization process proves crucial in reducing variability and enhancing the model’s overall efficacy during training.

Our study emphasizes tissue-level classification rather than patch-level analysis. While patch-level annotations (Hou et al., 2016; Mi et al., 2021) have gained popularity for cancer sub-type detection in digital pathology, relying solely on them has notable drawbacks. A primary concern is the substantial manual annotation effort required for this approach, involving the careful marking and annotation of individual patches within the WSIs, making the process time-consuming and labour-intensive. However, depending solely on patch-level analysis may result in overlooking crucial contextual information within the slide. By focusing exclusively on isolated patches, there is a risk of missing important features and spatial relationships between regions in the slide. This limitation can lead to inaccurate or incomplete detection, as the analysis may fail to capture the full extent of the disease and its characteristics. Moreover, a patch-level analysis may be susceptible to sampling bias (Hägele et al., 2020), as the selected patches may not adequately represent the entire slide, potentially causing the omission of critical regions of interest crucial for accurate cancer subtype identification.

Furthermore, the challenges of intratumoral heterogeneity pose a significant obstacle for patch-level cancer subtype detection. Cancer subtypes can exhibit spatial variability within a single tumor, with distinct regions displaying different cellular characteristics and molecular profiles. By relying solely on patch-level analysis, there is a risk of obtaining inconsistent or ambiguous results, as the selected patches may not fully capture the heterogeneity present within the tumor. This limitation can impact the reliability and accuracy of the cancer subtype detection, particularly when attempting to classify tumors with diverse intratumoral characteristics. We assessed the method’s effectiveness in detecting esophageal subtype carcinoma from H&E WSIs. We found that ML can accurately classify cancer subtypes in EC from H&E WSIs. Pathologists, who have a laborious, time-consuming, and easily misinterpreted diagnostic process, could benefit significantly from automated cancer subtype detection by having less work to do.

Recognizing that patch-level cancer subtype detection can be integrated with additional contextual information from WSI, such as slide-level annotations, can help overcome the limitations associated with patch-level analysis. By incorporating a broader perspective and considering the overall tissue architecture and spatial relationships within the slide, the accuracy and reliability of cancer subtype detection can be improved. Further research and development efforts are necessary to address these limitations and refine the performance of patch-level cancer subtype detection methods in the context of a digital pathology-based cancer diagnosis. Addressing data scarcity in tasks linked to histopathology is also crucial to maximizing the effectiveness of deep learning systems. High-quality and massive amounts of disease data with detailed tile annotations will be required to accelerate model building. DL models, are particularly suitable for large-scale data analysis (Jan et al., 2019), depending on the amount of data provided, making it challenging to apply them to real-world circumstances. Unfortunately, having enough data for DL models in most real-world circumstances is not feasible, especially in medical science (Zewdie et al., 2021). As an alternative, transfer learning has shown promising results for computer vision problems where only a few training samples are available (refer to Table 4). A feature extractor trained on various pathological datasets may produce better outcomes than our cancer sub-type-specific model.

**TABLE 4:** Comparison of studies with proposed architecture.

Study	Organ	Approach	Classifier	Dataset	Patients	Results
Wagner and Cosgrove (2023)	Uterus	Image level annotation	CNN	TCGA-UCEC	657	0.82 (AUC)
Zhao et al. (2023)	Lung	pixel-level annotation	CNN	Beijing Chest Hospital	523	0.80 (AUC)
Kumar A and Mubarak (2022)	Esophagus	Image level annotation	SVM	Kvasir dataset	1800	0.93 (ACC)
Wang et al. (2020)	Ovarian Cancer	Patch level annotation	Random Forest	Vancouver General Hospital	305	0.87 (ACC)
**Proposed Method**	**Esophagus**	**Image level annotation**	**FFNN**	**TCGA-ESCA**	**59**	**0.94 (ACC)**

Utilizing transfer learning with ResNet101 on histopathological imagery, our study achieved superior performance with a Feed Forward Neural Network, boasting 94% accuracy and 0.92 AUC. Support Vector Classifier and Logistic Regression followed closely with an 88% accuracy, outperforming Random Forest Classifier (RFC) at 82%, while Decision Tree (DT) and AdaBoost lagged with 64% accuracy. This approach was centred on examining H&E stained WSIs, renowned for their capacity to preserve intricate microscopic tissue properties, thereby providing a robust foundation for detailed cancer tissue analysis. Looking ahead, we propose the inclusion of WSIs from additional databases in future investigations. These datasets should ideally share resolutions and characteristics similar to those used in this study to ensure consistency and reliability in comparative analyses. A thorough assessment of these representations and their parameters could offer insightful perspectives on the accuracy and repeatability of diagnostic algorithms, especially considering the distinctive nature of realistic *versus* pathological imagery domains. To sum up, our research proves that ResNet101-based transfer learning outperforms other methods in analyzing histopathological images and presents new opportunities for improving cancer diagnosis techniques. By utilizing the capabilities of sophisticated neural network architectures, there is considerable potential to increase the precision and speed of cancer detection from histological images. In addition, we recognize the importance of addressing the interpretability of the proposed model and plan to incorporate explainable AI techniques as part of our future research.

## Data Availability

The original contributions presented in the study are included in the article/Supplementary material, further inquiries can be directed to the corresponding authors.
